# The IrlS2-IrlR2 two-component system is a global regulator of biofilm formation, stress adaptation, and virulence in *Burkholderia pseudomallei*

**DOI:** 10.1128/msphere.00744-25

**Published:** 2026-02-26

**Authors:** Fang Chen, Jian Luo, Yingjuan Wang, Shen Tian, Xun Kang, Nan Zhang, Wan Zheng, Wenting Li, Qianfeng Xia, Dai Kuang

**Affiliations:** 1 NHC Key Laboratory of Tropical Disease Control, School of Life Sciences and Medical Technology, Hainan Medical University 12455, Haikou, China; 2 Department of Infectious and Tropical Diseases, The Second Affiliated Hospital of Hainan Medical University 477165 , Haikou, China; 3 Department of Infectious Diseases, The First Affiliated Hospital of Anhui Medical University 36639, Hefei, China; Universita degli Studi di Napoli Federico II, Naples, Italy

**Keywords:** *Burkholderia pseudomallei*, two-component systems, biofilm formation, stress adaptation, virulence regulation

## Abstract

*Burkholderia pseudomallei*, which causes melioidosis, is an adaptable pathogen that survives in diverse environments. Two-component systems (TCSs) play pivotal roles in bacterial signal transduction and adaptation, yet the functions of most TCSs in *B. pseudomallei* remain poorly characterized. Here, we identified and functionally characterized a previously unstudied TCS, IrlS2-IrlR2, which shares partial homology with the IrlS-IrlR system but exhibits distinct regulatory roles. Knockout of *irlR2* (Δ*irlR2*) did not affect growth in nutrient-rich medium but led to enhanced biofilm formation, reduced motility, decreased siderophore production, and increased sensitivity to oxidative stress, all of which were restored in the complemented strain. The mutant also exhibited growth retardation under subinhibitory cobalt concentrations, despite unchanged MICs. In infection assays, Δ*irlR2* displayed impaired adhesion and cytotoxicity toward A549 cells and attenuated virulence in *Galleria mellonella*, with a higher median lethal dose than the wild type. Transcriptomic analysis revealed *hcp2* within the type VI secretion system 2 (T6SS-2) cluster, and multiple T3SS-3 genes were strongly downregulated, consistent with reduced intracellular survival, whereas T3SS-1 and T3SS-2 were upregulated, suggesting a dysregulated secretion system balance. Adhesion-related (*fimA*) and iron transport (*fhuBCDF*) genes were repressed, while oxidative stress-associated (*cydABX*) and nitrate reductase (*narIJHGK-nasA*) operons were induced, indicating altered regulation of iron homeostasis, redox balance, and nitrogen metabolism, which may reflect adaptive responses to environmental stress. Collectively, these results demonstrate that the IrlS2-IrlR2 system functions as a global regulator, integrating biofilm formation, stress adaptation, and virulence regulation, highlighting its role in the environmental resilience and pathogenic potential of *B. pseudomallei*.

**IMPORTANCE:**

*Burkholderia pseudomallei*, which causes melioidosis, poses a serious threat to human and animal health in tropical and subtropical regions worldwide. Classified as a tier 1 biothreat agent by the U.S. CDC and a category II pathogen in China, *B. pseudomallei* causes severe pneumonia and septicemia with case-fatality rates approaching 50%. Despite its medical and epidemiological significance, the regulatory mechanisms controlling its virulence and environmental persistence remain poorly understood. This study identifies IrlS2-IrlR2 as a previously uncharacterized two-component system (TCS) that acts as a global regulator integrating biofilm formation, stress adaptation, and virulence. Functional and transcriptomic analyses reveal that IrlS2-IrlR2 modulates secretion systems, iron homeostasis, and redox balance. These findings deepen our understanding of *B. pseudomallei* pathogenesis and highlight the role of TCS-mediated regulatory networks.

## INTRODUCTION

*Burkholderia pseudomallei* is a gram-negative, facultative intracellular pathogen that causes melioidosis, a potentially life-threatening disease in humans and animals in tropical and subtropical regions (1, 2). It is designated by the U.S. Centers for Disease Control and Prevention as a tier 1 select bioterrorism agent, reflecting its dual significance as both a clinical pathogen and a potential biothreat. Melioidosis presents with a wide spectrum of clinical manifestations, encompassing acute septicemic pneumonia, disseminated visceral abscesses, and local infections. *B. pseudomallei* exhibits remarkable adaptability to diverse environmental and host-associated niches, underpinned by its large two-chromosome genome (~7 Mb) encoding numerous virulence determinants such as lipopolysaccharide, capsule, flagella, and type III and type VI secretion systems (3, 4). These virulence factors contribute to both acute and chronic disease by promoting survival and immune evasion in diverse hosts and environments, yet the molecular mechanisms underlying their regulation remain largely undefined.

Two-component systems (TCSs) are ubiquitous signal transduction mechanisms in bacteria that enable rapid sensing and response to environmental signals by modulating physiological behaviors and molecular functions, thereby ensuring bacterial survival and adaptability under diverse conditions (5). A typical TCS consists of a membrane-associated histidine kinase and a cytoplasmic response regulator (6). By sensing environmental cues, these systems regulate gene expression at both transcriptional and post-transcriptional levels, influencing survival, antibiotic resistance, virulence, and metabolism (7, 8).

Comparative genomics has identified over 60 TCSs in *B. pseudomallei*, yet only a few have been functionally characterized (9). Well-studied examples include VirAG, which activates *hcp1* expression via T6SS-1 (10); NarXL, which responds to nitrate/nitrite and modulates biofilm formation and secondary metabolite synthesis (11); and IrlSR, which is linked to epithelial cell invasion and heavy metal resistance (12). Other TCSs, such as BprRS, MrgRS, NosKR, and RegAB, have also been implicated in regulating motility, environmental adaptation, redox balance, and virulence in *B. pseudomallei*, although their precise mechanisms remain to be fully elucidated (13–16).

In this study, we characterize a novel TCS, IrlS2-IrlR2, which shares 50% and 73% sequence identity with *irlS* and *irlR* of the IrlSR system, respectively (17). The IrlSR system has been implicated in cadmium and zinc resistance (12); however, the biological function of IrlS2-IrlR2 remains largely unexplored. To elucidate the role of IrlS2-IrlR2 in *B. pseudomallei*, particularly its involvement in virulence regulation, we constructed an *irlR2* knockout mutant (Δ*irlR2*) and compared its phenotypic traits to those of the wild-type (WT) strain. Phenotypic analyses focused on biofilm formation, motility, siderophore production, oxidative stress tolerance, and virulence in human alveolar basal epithelial A549 cells and the *Galleria mellonella* infection model. Additionally, transcriptomic profiling identified regulatory pathways modulated by IrlS2-IrlR2. Our findings suggest that IrlS2-IrlR2 significantly influences motility, biofilm formation, oxidative stress resistance, and virulence, potentially through direct or indirect regulation of type VI secretion system 2 (T6SS-2), T3SS, flagellar gene clusters, and nitrate reductase genes. This regulation affects not only the expression levels but also the activity of these systems, thereby contributing to the bacterial adaptability and pathogenicity of *B. pseudomallei*.

## RESULTS

### Generation of *irlR2* mutants reveals no effect on growth

**Fig 1:**
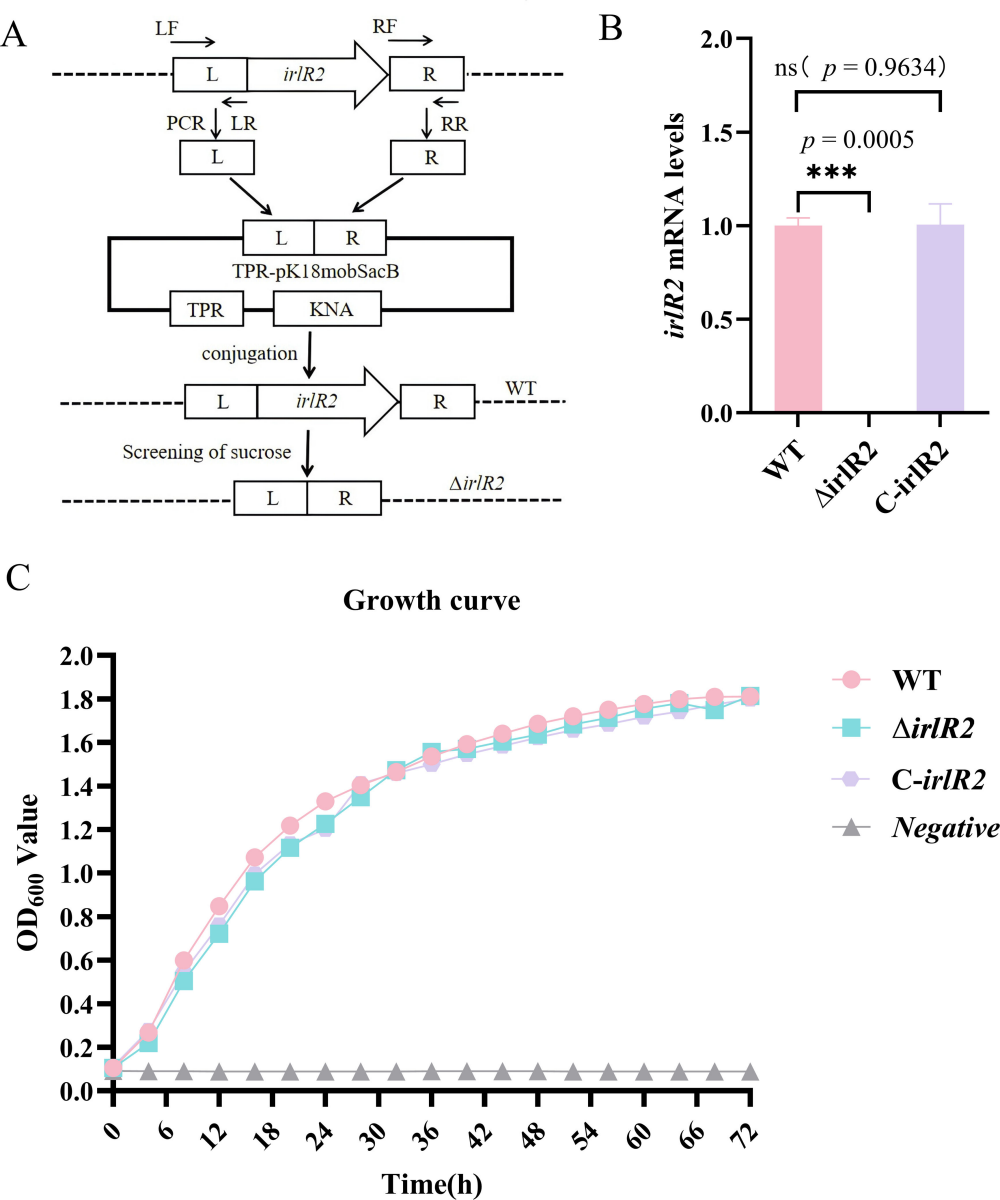
Construction of *irlR2* deletion and complementation strains and their effects on bacterial growth. (**A**) Schematic diagram illustrating the homologous recombination strategy used to construct the Δ*irlR2* mutant. (**B**) Quantitative analysis of *irlR2* expression levels among the wild-type (WT), Δ*irlR2*, and C-*irlR2* strains by qRT-PCR. (**C**) Growth curves of WT, Δ*irlR2*, and C-*irlR2* strains in Luria-Bertani broth, showing no significant differences. ns, not significant; ***P ≤ 0.001.

Given that *irlS2* encodes a transmembrane protein and may be essential for the growth of *B. pseudomallei*, we targeted *irlR2* for functional analysis. To investigate the role of *irlS2-irlR2* in regulating the biological properties and associated pathways of *B. pseudomallei*, we employed homologous recombination to generate an *irlR2* deletion mutant (Δ*irlR2*) in the *B. pseudomallei* HNBP001 background (Fig. 1A), and constructed its complemented derivative (C-*irlR2*) using the pUCP28T plasmid. We then extracted RNA from the WT, Δ*irlR2*, and C-*irlR2* strains and confirmed the successful deletion of *irlR2* by qRT-PCR (Fig. 1B).

We next monitored the growth kinetics of the WT, Δ*irlR2*, and C-*irlR2* strains in real time using a multimode microplate reader, recording OD_600_ values every 30 min over a 72-h period (Fig. 1C). No statistically significant differences were observed among the three strains, indicating that *irlR2* deletion does not impair the growth of *B. pseudomallei*.

### Deletion of *irlR2* promotes biofilm formation and reduces bacterial motility

**Fig 2:**
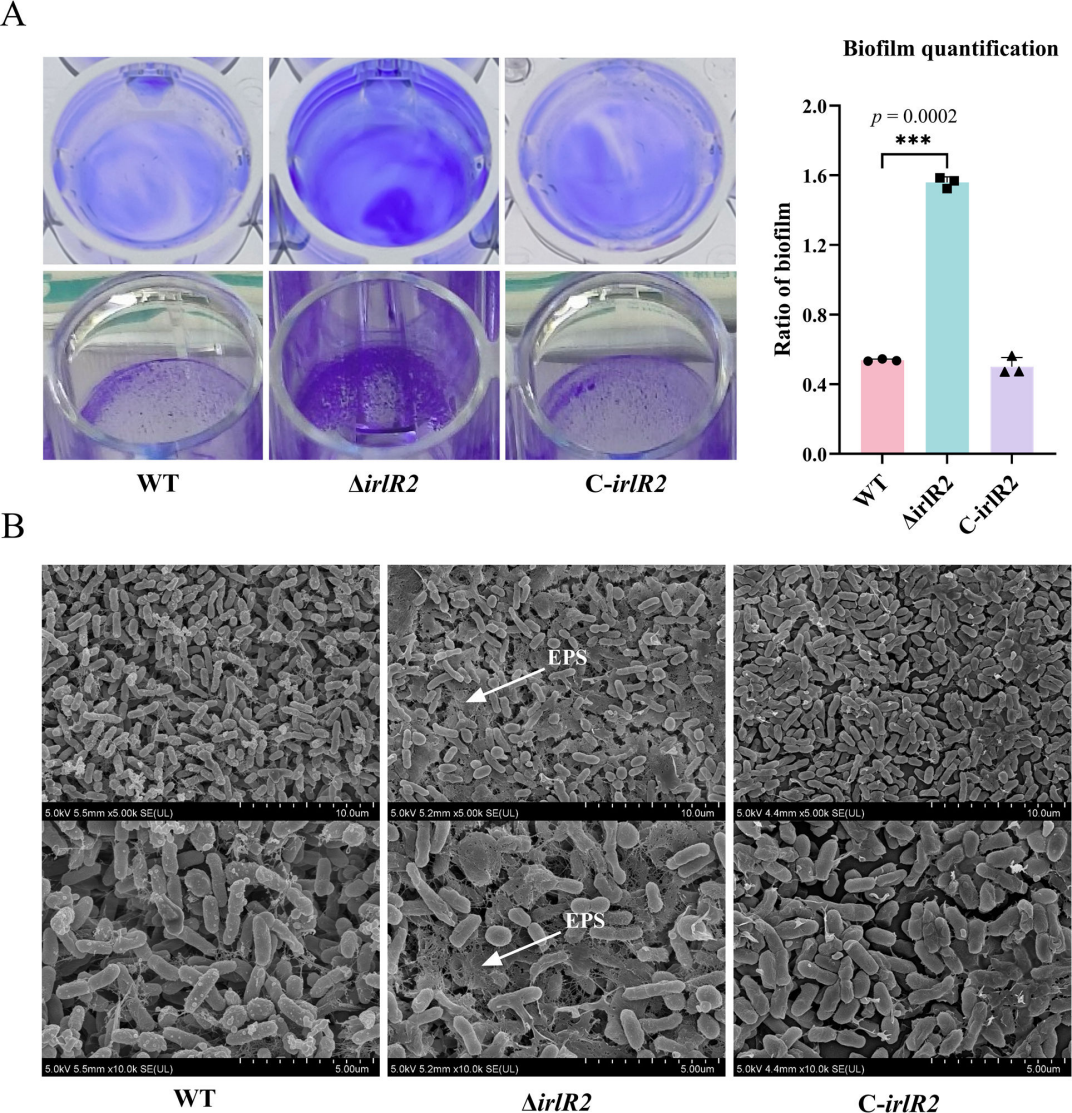
Effects of *irlR2* deletion on biofilm formation in *Burkholderia pseudomallei*. (**A**) Representative images and quantitative analysis of biofilms formed by WT, Δ*irlR2*, and C-*irlR2* strains. The top row of images shows biofilms after 33% acetic acid treatment, and the bottom row shows biofilms before treatment. ***P ≤ 0.001. (**B**) Scanning electron micrographs showing biofilm architecture on glass coverslips. Arrows indicate extracellular polymeric substances (EPS) surrounding the Δ*irlR2* mutant.

Biofilms represent a collective survival strategy employed by microorganisms, facilitating surface colonization and intercellular communication through the encapsulation of bacterial communities (18). To assess whether *irlR2* influences biofilm formation, we performed a polystyrene surface adhesion assay (19). The WT, Δ*irlR2* mutant, and complemented strains (C-*irlR2*) all developed typical biofilm structures at the air-liquid interface, characterized by floating pellicles and adherent rings on the tube walls. The Δ*irlR2* mutant produced a thicker biofilm at the air-liquid interface. Quantitative measurements confirmed a significant increase in biofilm biomass in the Δ*irlR2* mutant compared with the WT strain (*P* < 0.001) (Fig. 2A), and this phenotype was restored in the complemented strain. To further verify the abovementioned phenotypic observations, we conducted scanning electron microscopy (SEM) analysis on these three strains. The SEM images demonstrated that compared with the wild-type strain and the complemented strain C-*irlR2*, Δ*irlR2* exhibited increased biofilm formation, and the extracellular polymeric substances were visible around the colonies of Δ*irlR2* (Fig. 2B).

**Fig 3:**
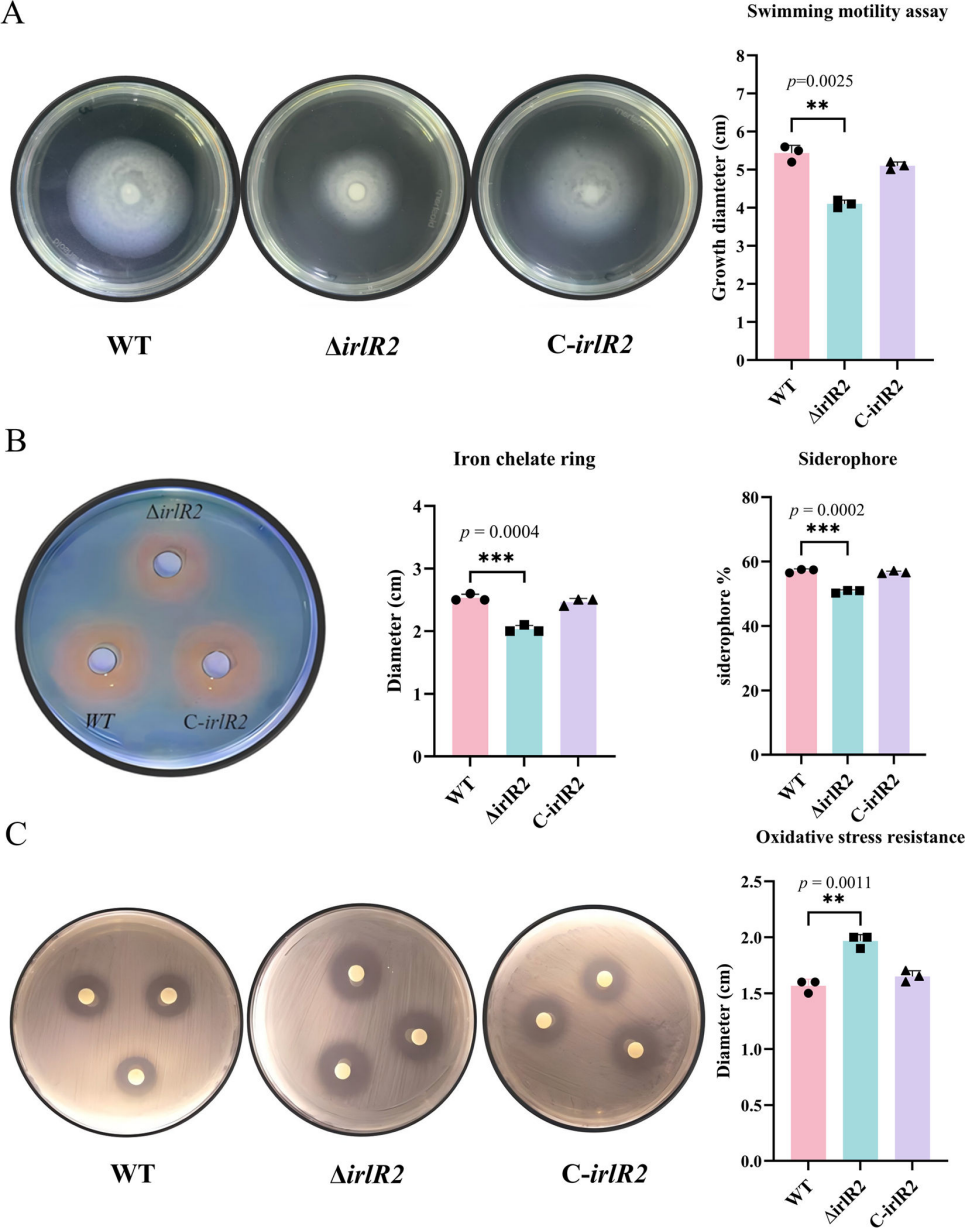
Effects of *irlR2* deletion on motility, siderophore production, and oxidative stress resistance. (**A**) Motility assay showing swimming diameters of WT, Δ*irlR2*, and C-*irlR2* strains after 24 h on 0.3% agar plates. (**B**) Siderophore production visualized on chrome azurol S (CAS) agar plates, where orange halos indicate siderophore secretion, and quantitatively measured using the CAS liquid assay. (**C**) Sensitivity to H_2_O_2_ measured by the disk diffusion assay. **P ≤ 0.01; ***P ≤ 0.001.

To determine whether *irlR2* affects motility in *B. pseudomallei*, we measured the migration diameters of the WT, Δ*irlR2* mutant, and C-*irlR2* strains after 24 h of incubation in 0.3% soft agar. The Δ*irlR2* mutant exhibited a significantly smaller motility diameter than the WT strain (*P* < 0.01), and motility was restored in the complemented strain (Fig. 3A). These findings indicate that *irlR2* deletion enhances biofilm formation but impairs motility in *B. pseudomallei*.

### Deletion of *irlR2* reduces the production of siderophores under iron-limited conditions

To evaluate the effect of the Δ*irlR2* mutation on siderophore production, we performed both qualitative and quantitative analyses using the chrome azurol S (CAS) assay (20). The WT, Δ*irlR2*, and C-*irlR2* strains all produced orange-yellow halos of varying intensities, indicating siderophore synthesis. However, the halo diameter of Δ*irlR2* was markedly smaller than that of the WT, whereas complementation restored it to WT levels (Fig. 3B). Quantitative CAS assays showed that siderophore production in the WT strain was approximately 58%, whereas Δ*irlR2* exhibited a reduction to about 50%. The C-*irlR2* strain displayed siderophore levels comparable to WT (Fig. 3B), confirming that deletion of *irlR2* significantly reduces total siderophore production in *B. pseudomallei* (*P* < 0.001).

### Deletion of *irlR2* weakens oxidative stress resistance

To investigate whether *irlR2* plays a role in the antioxidant defense of *B. pseudomallei*, we employed hydrogen peroxide (H_2_O_2_) as an oxidative stress agent and assessed bacterial resistance using the Kirby-Bauer disk diffusion assay (21). The results revealed that the Δ*irlR2* mutant exhibited a significantly larger inhibition zone diameter compared to the WT strain, with statistical significance (*P* < 0.01) (Fig. 3C). This finding indicates that the Δ*irlR2* mutant is more susceptible to oxidative stress, suggesting that *irlR2* is involved in modulating the antioxidant capacity of *B. pseudomallei*.

### The effects of heavy metals on the growth rate of Δ*irlR2*

**Fig 4:**
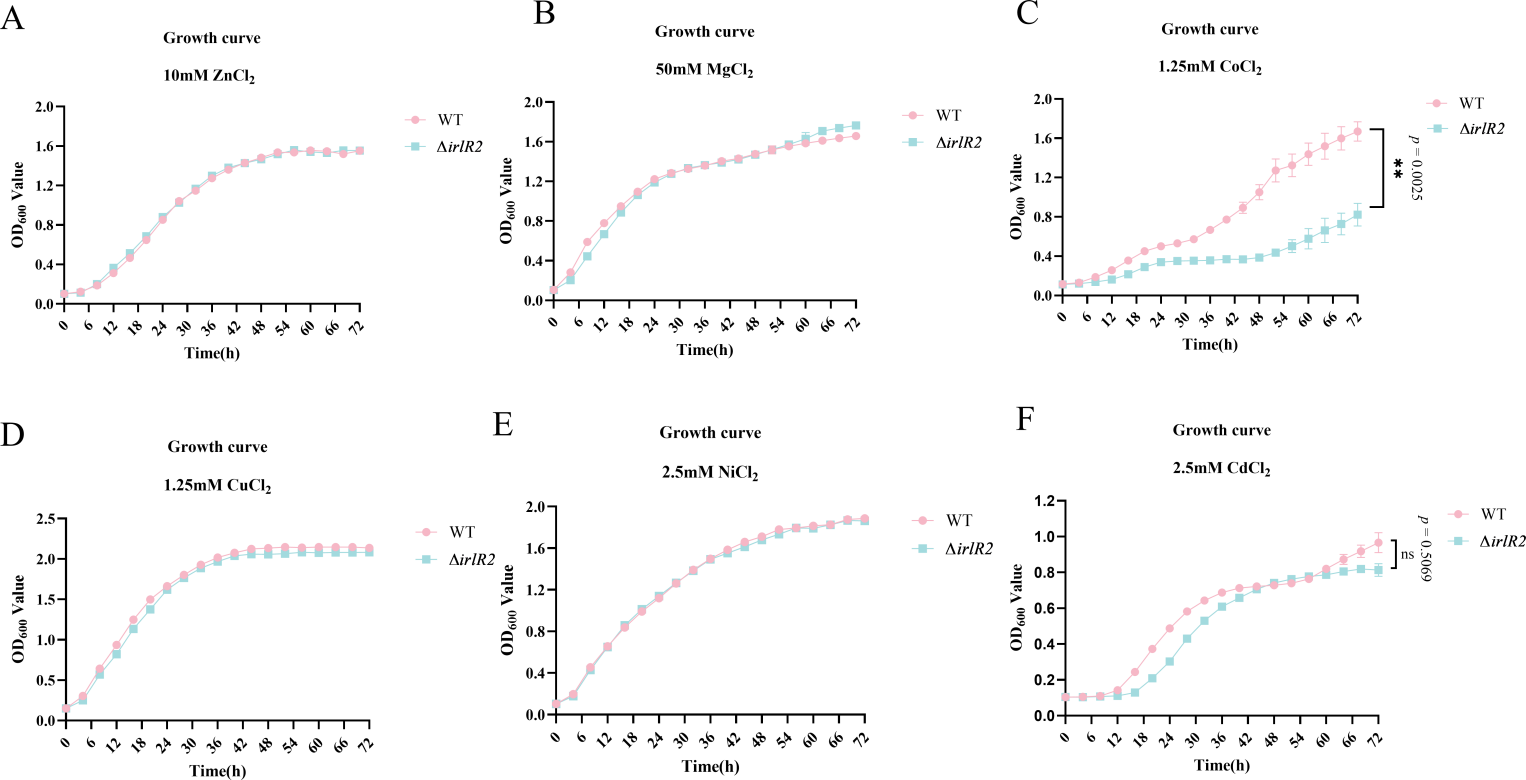
Effects of heavy metals on the growth of the Δ*irlR2* mutant. Growth kinetics of WT and Δ*irlR2* strains cultured in LB broth supplemented with subinhibitory concentrations of (**A**) ZnCl_2_, (**B**) MgCl_2_, (**C**) CoCl_2_, (**D**) CuCl_2_, (**E**) NiCl_2_, and (**F**) CdCl_2_. ns, not significant; **P ≤ 0.01.

It is well established that the TCS IrlS-IrlR system regulates eukaryotic cell invasion and resistance to heavy metals (12). To investigate whether IrlS2-IrlR2 also plays a role in heavy metal resistance in *B. pseudomallei*, we determined the minimum inhibitory concentrations (MICs) of zinc, cadmium, cobalt, nickel, copper, and magnesium for both the Δ*irlR2* mutant and the wild-type strain. Our results revealed no differences in MIC values between the two strains (Table S3). Subsequently, we assessed the impact of these metals on bacterial growth by comparing the growth kinetics of the Δ*irlR2* mutant and wild-type strains at sub-MIC concentrations. The Δ*irlR2* mutant exhibited delayed growth kinetics under cobalt exposure, with a significant difference (*P* < 0.01). No significant growth differences were detected for the other tested metals (Fig. 4).

### Deletion of *irlR2* reduces bacterial virulence in A549 cells and *G. mellonella* larvae

**Fig 5:**
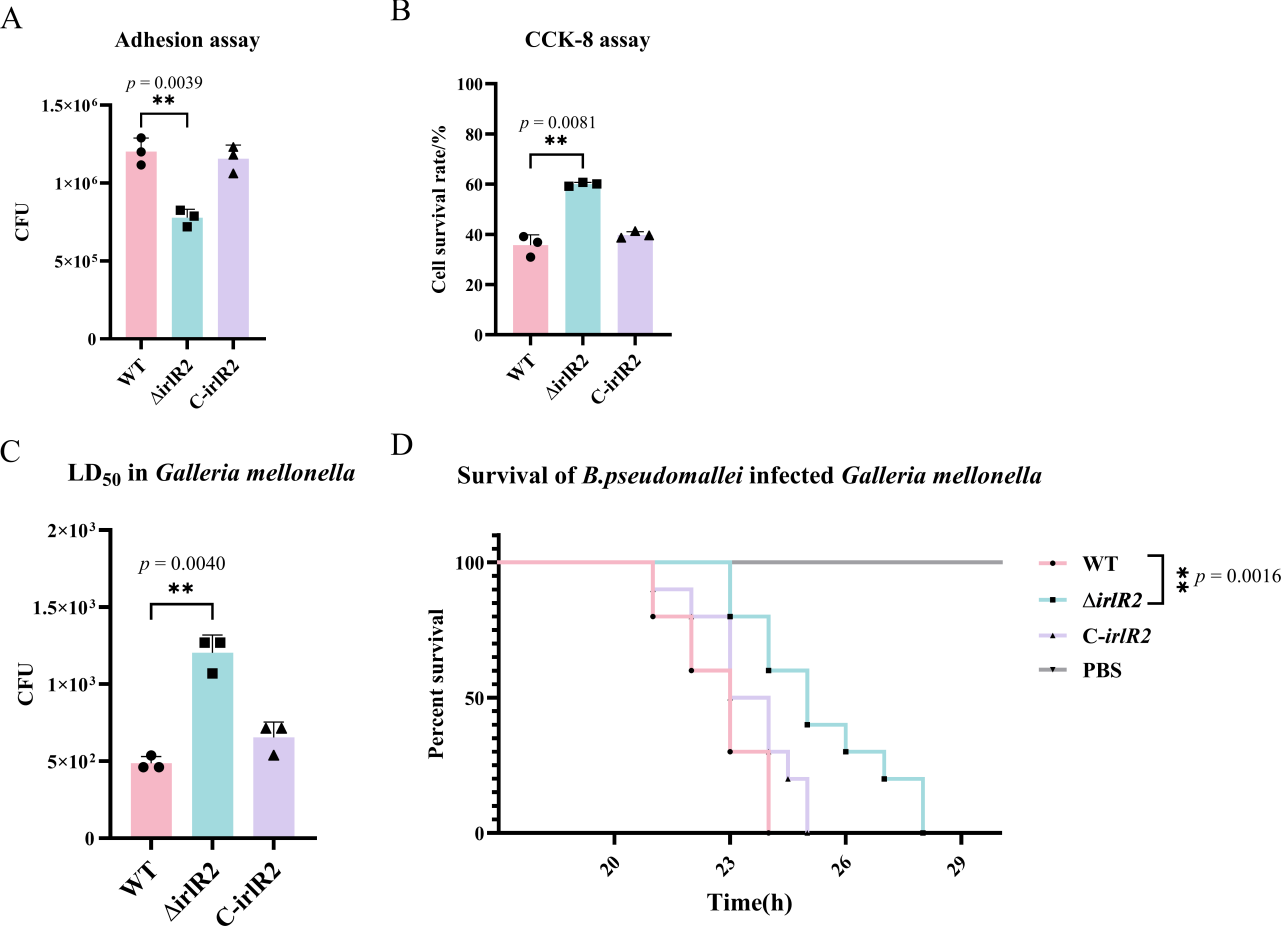
Deletion of *irlR2* reduces virulence in A549 cells and *Galleria mellonella* larvae. (**A**) Quantification of adhered bacteria following A549 infection. (**B**) Cytotoxicity toward A549 cells after 16 h of infection measured using the CCK-8 assay. (**C**) Median lethal dose (LD_50_) values determined 24 h post-infection in *G. mellonella*. (**D**) Survival curves of larvae infected with 1 × 10^3^ CFU of WT, Δ*irlR2*, or C-*irlR2* strains. **P ≤ 0.01.

Bacterial adhesion to host cells is essential for colonization, preventing clearance, and providing a competitive advantage over resident microbiota (22). To determine whether *irlR2* affects the adhesion capacity of *B. pseudomallei*, A549 human alveolar epithelial cells were used as an *in vitro* model. Following infection at a multiplicity of infection (MOI) of 10:1 for 2 h, colony-forming units (CFUs) were enumerated. The Δ*irlR2* mutant displayed a significantly reduced CFU count compared to the WT strain (*P* < 0.01) (Fig. 5A), indicating impaired adhesion and suggesting that *irlR2* positively regulates bacterial attachment to host cells.

To further assess the impact of *irlR2* deficiency on virulence, both A549 cells and *G. mellonella* larvae were employed as established infection models for *B. pseudomallei* (23, 24). Cytotoxicity toward A549 cells was measured using the CCK-8 assay following infection at an MOI of 10 for 16 h. The survival rate of A549 cells infected with Δ*irlR2* was approximately 60%, significantly higher than that of the WT and C-*irlR2* strains (WT about 37% and C-*irlR2* about 39%.) (*P* < 0.01) (Fig. 5B). In the *G. mellonella* infection model, the median lethal dose (LD_50_) of the Δ*irlR2* mutant was significantly higher than that of the WT strain (*P* < 0.01), and complementation restored virulence to near WT levels (Fig. 5C). Survival assays using 1 × 10^5^ CFU revealed that the WT strain caused about 100% mortality within 24 h, whereas Δ*irlR2* infection resulted in only 40% mortality (Fig. 5D). Together, these results demonstrate that *irlR2* is essential for full virulence of *B. pseudomallei* in both mammalian and insect infection models.

### Transcriptomic analysis reveals the regulatory impact of *irlR2* on multiple virulence- and stress-associated pathways

**Fig 6:**
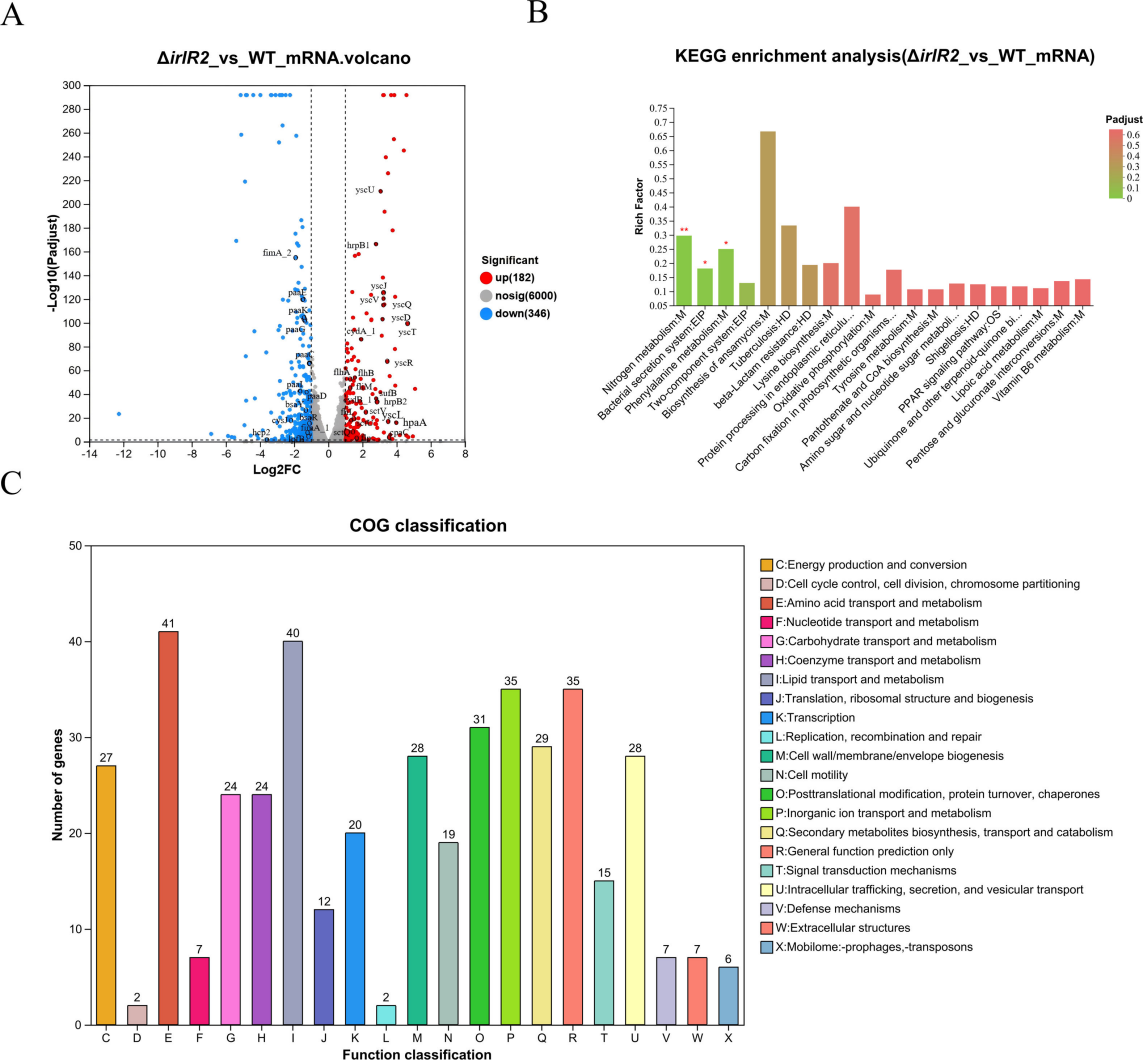
Transcriptomic analysis of the Δ*irlR2* mutant of *Burkholderia pseudomallei*. (**A**) Volcano plot showing differentially expressed genes (DEGs) in the Δ*irlR2* mutant compared with WT. (**B**) KEGG annotation revealed the number of DEGs in Δ*irlR2* compared with WT, which were enriched in various pathways. (**C**) Functional classification of DEGs based on Clusters of Orthologous Groups (COG) categories.

**Fig 7:**
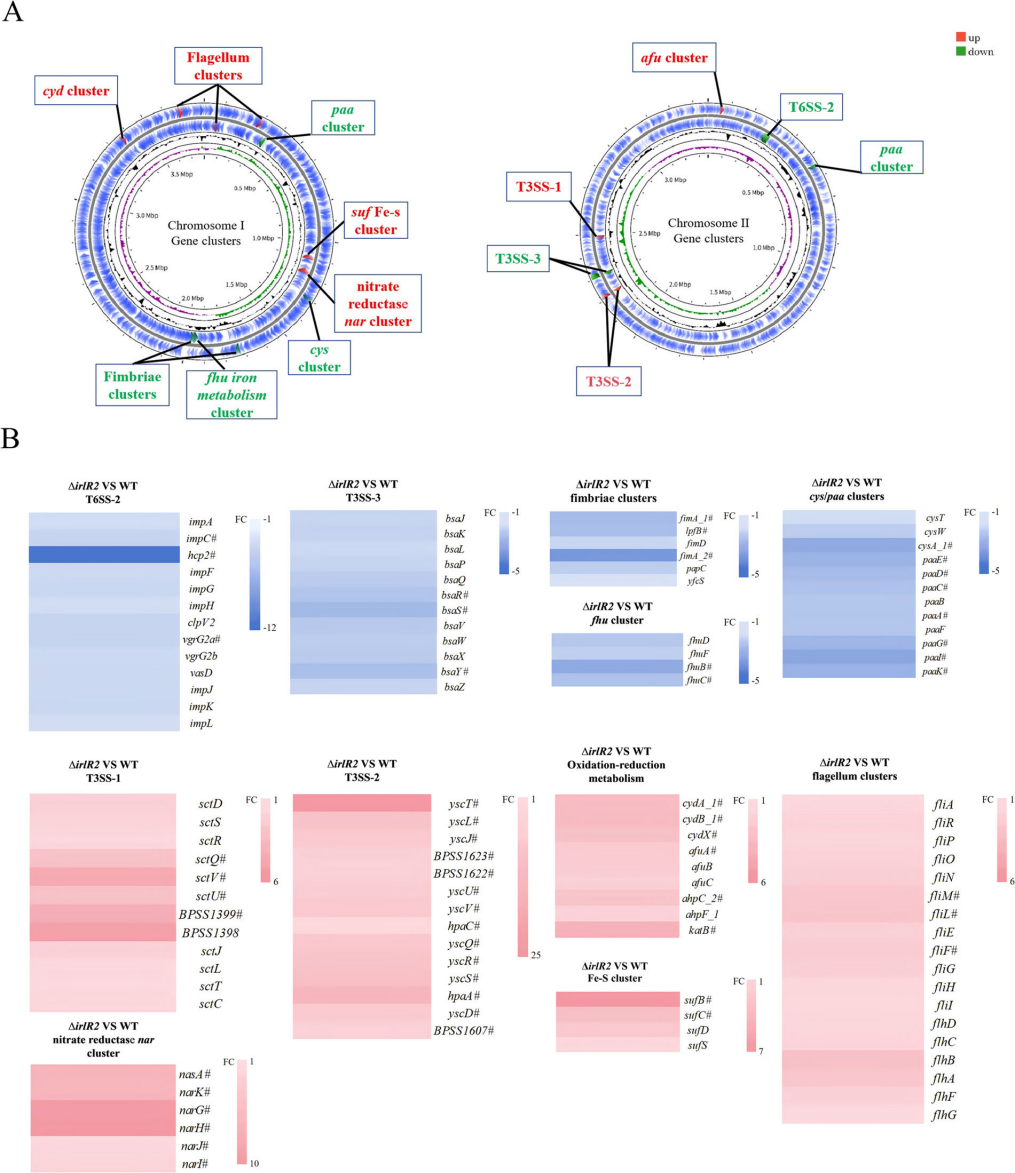
Genome-wide distribution and differential expression of functional gene clusters in Δ*irlR2*. (**A**) Circular genome map generated by Proksee showing the chromosomal distribution of differentially expressed clusters, including secretion systems, flagella, fimbriae, nitrogen metabolism, iron uptake, and secondary metabolism. Upregulated clusters are shown in red, and downregulated clusters are shown in green. (**B**) Heatmap of key differentially expressed gene clusters illustrating the average fold change (Δ*irlR2* versus WT) obtained from transcriptomic analysis. Values represent mean expression ratios from three independent biological replicates. “#” indicates statistically significant differential expression.

To investigate the molecular basis of the observed phenotypes, we performed RNA-Seq comparing Δ*irlR2* with WT. A total of 528 differentially expressed genes (DEGs) were identified, including 182 upregulated and 346 downregulated genes (Fig. 6A). Kyoto Encyclopedia of Genes and Genomes (KEGG) enrichment highlighted “bacterial secretion system,” “nitrogen metabolism,” and “phenylalanine metabolism” as the most significantly enriched pathways (Fig. 6B), while Clusters of Orthologous Groups (COG) analysis indicated that the main changes were an increase in the number of genes related to transport and metabolism, membrane-related functions, transcription, motility, and translation (Fig. 6C). Consistent with these findings, functional gene clusters associated with biofilm formation, adhesion, and secondary metabolism were mainly located on chromosome I, whereas virulence-related clusters, particularly secretion systems, were enriched on chromosome II (Fig. 7A).

At the level of virulence determinants, T6SS-2 was strongly repressed (*hcp2* downregulated 11.9-fold), and several T3SS-3 genes (*bsaR*, *bsaS*, and *bsaY*) were significantly suppressed, consistent with reduced intracellular survival. In contrast, T3SS-1 and T3SS-2 were upregulated, likely reflecting compensatory or dysregulated activation. Adhesion-related genes, including *fimA*, were also downregulated, aligning with impaired adhesion (Fig. 7B).

Genes involved in iron and oxidative stress responses were dysregulated: *fhuBCDF* (iron transport) was downregulated, whereas *sufBCD* (iron-sulfur assembly) and *cydABX* (cytochrome bd oxidase) were upregulated. Antioxidant enzymes *katB* and *ahpCF* were also induced, consistent with reduced siderophore production and increased reactive oxygen species (ROS) sensitivity. Beyond virulence and stress responses, metabolic genes such as *paa* (phenylacetate degradation) and *cys* (cysteine biosynthesis) were downregulated, while the nitrate reductase operon (*narIJHGK-nasA*) was upregulated, implicating IrlR2 in central metabolism and nitrogen utilization. Flagellar biosynthesis genes, including *fliA*, were globally upregulated despite reduced motility, suggesting regulatory imbalance rather than transcriptional deficiency.

Together, these results indicate that IrlS2-IrlR2 broadly regulates secretion systems, adhesion, iron homeostasis, oxidative stress defenses, and central metabolism, with its loss leading to extensive transcriptional rewiring that explains the pleiotropic phenotypes of Δ*irlR2*.

## DISCUSSION

TCSs are ubiquitous in bacteria and play essential roles in coordinating cellular responses to environmental changes. Given the abundance of TCSs encoded in the *B. pseudomallei* genome, these signaling systems are thought to constitute pivotal regulatory networks that govern diverse physiological processes, including antimicrobial resistance, virulence, and adaptation to fluctuating environmental conditions. Despite the large number of predicted TCSs in *B. pseudomallei*, only a limited subset has been experimentally characterized. Here, we identify IrlS2-IrlR2 as a previously uncharacterized TCS that exerts broad regulatory effects on virulence-related traits, including biofilm formation, motility, siderophore production, oxidative stress tolerance, and host infection capacity.

A striking phenotype of the Δ*irlR2* mutant was the trade-off between enhanced biofilm formation and impaired motility. This shift likely reflects an adaptive strategy: motility in the wild-type strain promotes environmental dissemination, whereas enhanced biofilm formation in the Δ*irlR2* mutant compensates for reduced dispersal, supporting survival under static conditions. Similar trade-offs have been reported for the FixLJ system in the *Burkholderia cepacia* complex, where deletion leads to stronger biofilms but reduced swimming motility and attenuated virulence (25). Intriguingly, transcriptomic data revealed upregulation of multiple flagellar genes, including *fliA* in Δ*irlR2*, yet functional motility decreased. This mismatch suggests that the defect stems not from structural deficiencies but from dysregulated expression timing, protein stoichiometry, or energy allocation. One possible explanation is the reduced expression of the nitrate reductase cluster (*narIJHG-nasA*), which may lower nitric oxide (NO) production. As NO activates guanylate cyclase and elevates c-di-GMP, its reduction may shift intracellular signaling, promoting biofilm formation at the expense of motility (26–28). These findings echo the role of NarX-NarL in *B. pseudomallei*, which senses nitrate/nitrite and regulates biofilm formation, metabolism, and host survival (11).

In addition to altered motility-biofilm balance, Δ*irlR2* showed impaired resistance to oxidative stress and reduced siderophore production. While the decrease in siderophore production was modest in magnitude, it was reproducible and coincided with increased oxidative stress sensitivity, supporting a biologically relevant disruption of iron-redox homeostasis. These phenotypes are consistent with the transcriptomic downregulation of the *fhuACDB* operon, which mediates Fe^3+^-siderophore uptake (29, 30), and the compensatory upregulation of the *suf* cluster involved in iron-sulfur assembly (31, 32). This pattern suggests disrupted iron homeostasis and a stress-induced adaptive response. Because iron metabolism and redox regulation are tightly linked, these alterations likely compromise the mutant’s ability to maintain iron homeostasis and oxidative stress defenses. Comparable relationships have been demonstrated in *Pseudomonas aeruginosa*, where BfmRS TCS mutants produce fewer siderophores and are attenuated in infection models (33). Within *B. pseudomallei*, the RegAB system serves as a master redox regulator coordinating anaerobic metabolism and virulence (16), whereas NosP functions as a nitric oxide sensor modulating biofilm formation, motility, and nitrosative stress responses (15). These parallels suggest that IrlS2-IrlR2 contributes to the broader redox-iron-virulence regulatory network that underpins *B. pseudomallei* pathogenesis.

Regulation of secretion systems also emerged as a major downstream outcome of IrlR2. Transcriptomics revealed strong repression of T6SS-2, including about 12-fold downregulation of *hcp2*. In *Burkholderia thailandensis*, T6SS-2 is linked to ROS defense and metal ion transport (34), suggesting its repression in Δ*irlR2* weakens oxidative stress tolerance and virulence. In contrast, type III secretion systems displayed divergent regulation: T3SS-1 and T3SS-2 were markedly upregulated, while T3SS-3 was repressed. Currently, T3SS-1 and T3SS-2 have not been shown to contribute to virulence in mammalian hosts, though they are homologous to the *hrp2* system in plant pathogens and functional in non-mammalian models (35). Since T3SS-3 is essential for endosomal escape and full virulence in animal models (36), its downregulation likely explains the reduced cytotoxicity and attenuated infection observed in A549 cells and *G. mellonella*. In this context, the attenuation observed in the *G. mellonella* model was moderate, indicating that IrlS2-IrlR2 contributes to, but does not solely determine, virulence in this host. These findings suggest that IrlS2-IrlR2 fine-tunes secretion system expression to optimize infection efficiency across distinct host environments. Furthermore, transcriptional downregulation of fimbrial genes, including *fimA*, which encodes the major subunit of type I fimbriae, provides a molecular basis for the observed reduction in adhesion and virulence (37).

Although homologous to the canonical IrlS-IrlR system previously associated with heavy metal resistance (12), IrlS2-IrlR2 appears functionally distinct. Δ*irlR2* exhibited no differences in MICs for zinc, cadmium, cobalt, nickel, copper, or magnesium. However, growth delays under sub-MIC concentrations of cadmium and cobalt suggest that IrlS2-IrlR2 may influence growth dynamics under specific stress conditions, perhaps indirectly through effects on redox and iron balance. This functional divergence highlights the specialization of TCS paralogs within the *B. pseudomallei* genome.

This study has several limitations. First, we focused on the response regulator IrlR2, leaving the role of its cognate sensor kinase irlS2 unresolved. The environmental signals that activate IrlS2 remain to be identified, as do the membrane or periplasmic factors that modulate its sensing specificity. Second, potential crosstalk or compensatory interactions between the IrlS2-IrlR2 system and other TCS or global regulators were not explored, although such interactions may underlie the pleiotropic phenotypes observed. Third, while transcriptomic data implicate IrlR2 in regulating secretion, iron metabolism, and adhesion genes, further work is required to determine whether these effects result from direct promoter binding or indirect regulatory cascades.

**Fig 8:**
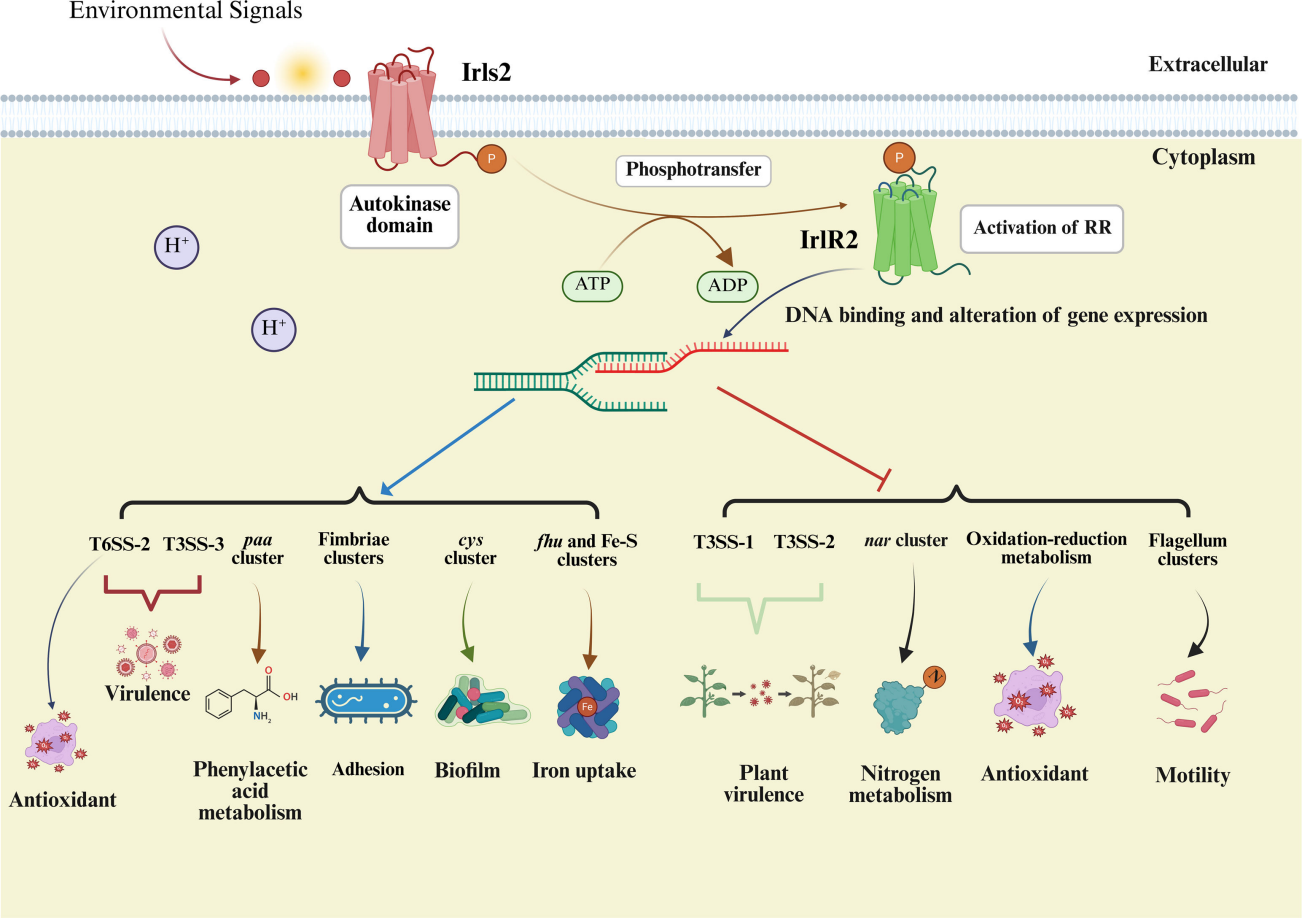
Proposed regulatory model of the IrlS2-IrlR2 two-component system in *Burkholderia pseudomallei*. The model depicts a putative signaling cascade in which the sensor kinase IrlS2 senses environmental cues, undergoes autophosphorylation, and transfers the phosphate group to the response regulator IrlR2, following the canonical mechanism of two-component systems. Activated IrlR2 is proposed to modulate the transcription of multiple functional gene clusters involved in secretion systems, adhesion, biofilm formation, iron uptake, nitrogen metabolism, oxidative stress response, and motility. Whether IrlR2 regulates these targets directly or indirectly remains to be determined. Arrows indicate regulatory trends inferred from transcriptomic data, with blue representing upregulation and red indicating downregulation.

In conclusion, we identify IrlS2-IrlR2 as a novel TCS that integrates biofilm-motility dynamics, iron acquisition, oxidative stress defense, secretion systems, and adhesion in *B. pseudomallei* (Fig. 8). By coordinating these diverse functions, IrlS2-IrlR2 enhances bacterial adaptability and pathogenic potential in fluctuating environmental and host conditions. This work broadens our understanding of TCS-mediated regulation in *Burkholderia* and provides a foundation for future research to define the molecular circuitry and environmental signals that drive IrlS2-IrlR2 activity in melioidosis pathogenesis.

## MATERIALS AND METHODS

### Bacterial strains, plasmids, primers, and growth conditions

The strains and plasmids used in this study are listed in Table S1, and primer sequences are provided in Table S2. All strains were cultured in Luria-Bertani (LB) broth at 37°C or on LB agar supplemented with appropriate antibiotics. *B. pseudomallei* HNBP001 was obtained from our laboratory collection, and its genome sequence has been deposited in NCBI (accession no. GCF_004842085.1).

### Construction of gene knockout strain

The ∆*irlR2* mutant was generated via homologous recombination (38). Genomic DNA from the WT strain HNBP001 served as the template to amplify the upstream and downstream homologous arms of *irlR2*. The amplified fragments were cloned into suicide vector TPR-pK18mobSacB using a seamless cloning kit (Vazyme Biotech, China). Recombinant plasmids were first transformed into *Escherichia coli* DH5α by heat shock, selected on trimethoprim-supplemented plates, and verified by PCR. The verified plasmid was subsequently transformed into *E. coli* S17-1λpir and conjugated into WT *B. pseudomallei* HNBP001. Counterselection on LB plates containing 15% sucrose was used to isolate colonies that had undergone double crossover recombination. Successful deletion of *irlR2* was confirmed by PCR and sequencing, resulting in the Δ*irlR2* mutant strain.

### Construction of the complementation strain

For complementation, *irlR2* was amplified from WT genomic DNA and cloned into the shuttle vector pUCP28T to generate pUCP28T-*irlR2*. Competent ∆*irlR2* cells were prepared by washing exponential-phase cells three times with ice-cold sterile water and resuspending in 10% glycerol. Approximately 10 μL of the plasmid DNA was mixed with 100 μL of competent cells, incubated on ice for 30 min, and electroporated at 2.5 kV. Following recovery in LB at 37°C for 3 h, cells were plated on LB agar containing 100 μg/mL trimethoprim and 100 μg/mL gentamicin. Colonies were verified by PCR and qRT-PCR, yielding the complemented strain C-*irlR2*.

### Determination of bacterial growth curves

Growth curves were determined using a multifunctional microplate reader (BioTek Synergy H1, USA) (39). Overnight cultures of WT, Δ*irlR2*, and C-*irlR2* strains were inoculated into fresh LB broth at a 1:100 ratio and grown at 37°C, 220 rpm until reaching OD_600_ = 0.5 ± 0.02. The cultures were then diluted 1:50 in LB broth, and OD_600_ values were recorded at regular intervals. Each strain was tested in triplicate, and results were expressed as mean ± standard deviation (SD).

### Biofilm formation assay

Biofilm formation was assessed in LB broth as previously described with minor modifications (40). WT, Δ*irlR2*, and C-*irlR2* strains were incubated statically at 37°C for 72 h. Biofilms were stained with 1% crystal violet, washed with PBS, and dissolved in 33% acetic acid. Absorbance at 595 nm was measured to quantify biofilm biomass. Each experiment was independently performed in triplicate.

### SEM of biofilms

Biofilms were formed on glass coverslips placed in 12-well plates containing 2 mL of bacterial suspension (OD_600_ = 0.1) and incubated statically at 37°C for 72 h. Coverslips were rinsed with PBS and fixed overnight in 4% (vol/vol) glutaraldehyde at 4°C. After washing, samples were dehydrated through an ethanol gradient (30%, 50%, 70%, 90%, and 100%), dried, gold-coated, and examined using a scanning electron microscope.

### Motility assays

The culture and passage methods of *B. pseudomallei* were the same as described previously. Bacterial suspensions were adjusted to OD_600_ = 0.1, and 2 μL was inoculated onto 0.3% LB agar plates. Plates were incubated at 37°C for 24 h, and the diameter of the motility zone was measured. Three biological replicates were included per strain.

### Iron acquisition assays

*B. pseudomallei* strains cultured overnight in LB broth were transferred to fresh LB containing 200 μg/mL 2,2′-dipyridyl for iron starvation and subcultured to an OD_600_ of 0.5. Then, 100 μL aliquots were spotted onto CAS-Fe(III) agar plates supplemented with cetyltrimethylammonium bromide (Hope Biotechnology Co., Ltd., China) and incubated at 37°C overnight. The formation of an orange halo around the colonies indicated siderophore production (20). For quantitative analysis, culture supernatants were mixed 1:1 with CAS reagent (Bioisco Biotech, China) and incubated in the dark for 2 h, and absorbance was measured at 630 nm. Siderophore unit (SU) values were calculated as SU = 1 − *A* / Ar (41), where *A* is the sample absorbance and Ar is the reference absorbance of the control medium. All experiments were independently performed in triplicate.

### Oxidative stress tolerance assays

Hydrogen peroxide sensitivity was tested by the paper disk diffusion method (42). Bacterial suspensions were adjusted to an OD_600_ of 0.5, diluted 1:5 with sterile PBS, and spread evenly on Mueller-Hinton (MH) agar. Sterile paper disks (6 mm) were placed on the surface and spotted with 10 μL of 0.15% H_2_O_2_. Plates were incubated at 37°C for 18 h, and inhibition zones were measured. Each experiment was performed in triplicate.

### MIC and growth assays under metal stress

Single colonies of each strain were inoculated into LB broth and incubated overnight at 37°C with shaking. Cultures were subcultured 1:100 into fresh LB medium and grown to logarithmic phase. The bacterial suspension was adjusted to 0.5 McFarland units, and 50 μL was inoculated into 96-well plates containing serial twofold dilutions of metal salts in MH broth. Plates were sealed and incubated at 37°C for 18 h, and the MIC was determined as the lowest concentration showing no visible growth. For sub-MIC growth assays, each strain was diluted 1:50 into LB supplemented with metals at 1/2 MIC concentrations, and OD_600_ values were measured over time using a microplate reader. Data represent mean ± SD from three biological replicates.

### Adhesion and cytotoxicity assay

A549 non-small lung cancer cells (ATCC) were infected with the WT, Δ*irlR2*, or C-*irlR2* strains at an MOI of 10 for 2 h. After infection, cells were washed three times with PBS to remove non-adherent bacteria and lysed with 1 mL of 0.1% Triton X-100 for 30 min to release attached bacteria. Lysates were serially diluted, plated on LB agar, and incubated at 37°C for 24 h to enumerate adherent CFUs.

Cytotoxicity toward A549 cells was measured using a CCK-8 kit (43). Cells were seeded into 96-well plates at a density of 2 × 10^4^ cells per well and incubated overnight. On the following day, cells were washed twice with sterile PBS and maintained in DMEM without phenol red. Mid-logarithmic phase suspensions of strains (WT, Δ*irlR2*, and C-*irlR2*) were added at an MOI of 10, and infection proceeded for 16 h. After washing to remove extracellular bacteria, CCK-8 solution was added and incubated for 60 min. Absorbance at 450 nm was measured with an ELISA reader to determine cell viability. All assays were performed using three independent biological replicates, with technical replicates included within each experiment.

### *G. mellonella* infection assay

Virulence was assessed using the *G. mellonella* larva infection model with slight modifications (24). Mid-log-phase cultures of WT, Δ*irlR2*, and C-*irlR2* strains were harvested, washed, and adjusted to defined concentrations. Serial 10-fold dilutions (10^2^–10^7^ CFU per larva) were injected to determine the LD_50_ by Probit regression based on pooled results from three independent experiments. Survival curves were then generated using a fixed infection dose (1 × 10^3^ CFU per larva), and differences among groups were analyzed by the log-rank (Mantel-Cox) test (GraphPad Prism, San Diego, USA). Each group contained 10 larvae, and all assays were performed in triplicate.

### Transcriptome analysis by RNA-Seq

WT and Δ*irlR2* strains were cultured to mid-logarithmic phase, and total RNA was extracted using a bacterial RNA extraction kit (Vazyme Biotech). Three biological replicates were included per strain. RNA-seq was performed by Shanghai Majorbio Bio-Pharm Technology Co., Ltd., using the Illumina platform. DEGs were defined by an adjusted *P* value of <0.05 (44). Functional and pathway enrichment analyses were performed using the KEGG and COG databases. Data visualization, including heat maps and genome-wide distribution of DEGs, was generated using Majorbio Cloud Platform (45), SRplot (46), and Proksee (47).

### Statistical analysis

Statistical analyses were performed using GraphPad Prism 10. Data are presented as mean ± SD from at least three independent biological replicates. Paired *t*-tests were applied for within-group comparisons, unpaired *t*-tests for between-group comparisons, and one-way ANOVA for multiple groups, followed by Dunnett’s multiple comparison test where appropriate. Statistical significance was defined as *P* ≤ 0.05 (*), *P* ≤ 0.01 (**), and *P* ≤ 0.001 (***).

## Data Availability

Raw sequencing data generated in this study have been deposited in the NCBI SRA database under BioProject accession number PRJNA1340332.

## SUPPLEMENTAL MATERIAL

Supplemental tables msphere.00744-25-s0001.docx Tables S1 to S3.

ASM does not own the copyrights to Supplemental Material that may be linked to, or accessed through, an article. The authors have granted ASM a non-exclusive, world-wide license to publish the Supplemental Material files. Please contact the corresponding author directly for reuse.
