# Supplementary material for: The IrlS2-IrlR2 two-component system is a global regulator of biofilm formation, stress adaptation, and virulence in Burkholderia pseudomallei
Source: mSphere. 2026 Feb 26;11(3):e00744-25. doi: 10.1128/msphere.00744-25 (PMC13037405; doi:10.1128/msphere.00744-25)
Supplement: Supplemental tables — Tables S1 to S3. [file msphere.00744-25-s0001.docx]

**The IrlS2-IrlR2 two-component system is a global regulator of biofilm formation, stress adaptation, and virulence in *Burkholderia pseudomallei***

Supplementary data

Table S1. Strains and plasmids used in this study.

Table S2. Oligonucleotides used in this study.

Table S3. Minimum inhibitory concentrations (MICs) of metal ions.

Table S1. Strains and plasmids used in this study.

| Strain/plasmid | Description | Source or Reference |
| --- | --- | --- |
| HNBP001 WT | Hainan *B. Pseudomalle*i representative strain, used as the wild-type strain | Xia QF, 2019 ( PMID: 31488524) |
| HNBP001 Δ*irlR2* | *irlR2* deleted via homologous recombination | This study |
| HNBP001C-*irlR2* | Complemented strain carrying *irlR2* in trans | This study |
| *E. coli* DH5α | Cloning host used for plasmid propagation and construction | Beijing Zhuangmeng Biotechnology Co., Ltd. |
| *E. coli* S17-1 λpir | Donor strain used for conjugative transfer of suicide plasmids; Sm^r^ Tp^r^Pb^s^ Km^r^ | Beijing Zhuangmeng Biotechnology Co., Ltd. |
| TPR-pK18mobSacB-Δ*irlR2* | Suicide vector containing upstream and downstream flanking regions of *irlR2*; Tp^r^Km^r^ | This study |
| pUCP28T-*irlR2* | Complementation plasmid carrying *irlR2* under native promoter; Tp^r^ | This study |

Table S2. Oligonucleotides used in this study.

| Primers | Sequence (5’→3’) | Purpose / Features |
| --- | --- | --- |
| Δ*irlR2*-LF | gaattcgagctcggtacccgCGTAAGCGGCGATCCAGTAG | Amplification of upstream arm of *irlR2* |
| Δ*irlR2*-LR | tgagtttgaaGCCGTGCGAATCGTGTGC |  |
| Δ*irlR2*-RF | tcgcacggcTTCAAACTCATTAAGAACGC | Amplification of downstream arm of *irlR2* |
| Δ*irlR2*-RR | aagcttgcatgcctgcaggtACATCGATAGCAAGGTCATC |  |
| pK18mobSacB-F | ACCTGCAGGCATGCAAGC | Linearization of the TPR-pK18mobSacB plasmid |
| pK18mobSacB-R | CGGGTACCGAGCTCGAATT |  |
| Δ*irlR2*-F | CTGCTCGAGATCTGATCGGC | Validation of the deletion of *irlR2* |
| Δ*irlR2*-R | TGCTCCGCTTTTCTACTCCG |  |
| p-*irlR2*-F | agctcggtacccggggatccGACGCCGGCGGGCATCGC | Cloning of *irlR2* and its native promoter |
| p-*irlR2*-R | gcctgcaggtcgactctagATCATGCACACGATTCGCACG |  |
| pUCP-F | TCTAGAGTCGACCTGCAGGCA | Linearization of pUCP28T plasmid |
| pUCP-R | GGATCCCCGGGTACCGAG |  |
| M13-F | CAGGAAACAGCTATGAC | Validation of cloning of the flanking arms of *irlR2* in pK18mobSacB-Δ*irlR2* and *irlR2* complementation in pUCP28T-*irlR2* |
| M13-R | GTAAAACGACGGCCAGT |  |
| 16S rRNA-F | GTGGGGAATTTTGGACAATG | Reference gene for qRT-PCR normalization |
| 16S rRNA-R | CCGGGTATTAGCCAGAATGA |  |
| *irlR2*-qF | GCTTCGCGAGATAGTCGTCA | qRT-PCR of *irlR2* |
| *irlR2*-qR | ATCAGCGGTCTGCATCAGTT |  |

*Note:* Underlined sequences indicate restriction enzyme sites; lowercase letters represent homologous overlap regions used for seamless cloning.

Table S3. Minimum inhibitory concentrations (MICs) of metal ions

| Metal Salt | MIC (mM)  WT ∆*irlR2* | |
| --- | --- | --- |
| Cadmium chloride (CdCl_2_) | 5 | 5 |
| Cobalt chloride (CoCl_2_) | 2.5 | 2.5 |
| Nickel chloride (NiCl_2_) | 5 | 5 |
| Copper chloride (CuCl_2_) | 2.5 | 2.5 |
| Zinc chloride (ZnCl_2_) | 20 | 20 |
| Magnesium chloride (MgCl_2_) | 100 | 100 |
